# Cri-du-chat syndrome mimics Silver-Russell syndrome depending on the size of the deletion: a case report

**DOI:** 10.1186/s12920-018-0441-z

**Published:** 2018-12-27

**Authors:** Yerai Vado, Javier Errea-Dorronsoro, Isabel Llano-Rivas, Nerea Gorria, Arrate Pereda, Blanca Gener, Laura Garcia-Naveda, Guiomar Perez de Nanclares

**Affiliations:** 1 Rare Diseases Research Group. Molecular (Epi)Genetics Laboratory, BioAraba Health Research Institute, OSI Araba University Hospital, Vitoria-Gasteiz, Araba, Spain; 2 Laboratory of Pharmacy and Pharmaceutical Technology, Faculty of Pharmacy, University of the Basque Country (UPV/EHU), Vitoria-Gasteiz, Araba Spain; 3 Service of Genetics, BioCruces Bizkaia Health Research Institute, Hospital Universitario Cruces, Barakaldo, Bizkaia Spain; 4 Service of Pediatric Neurology, BioAraba Health Research Institute, Hospital Universitario Araba-Txagorritxu, Vitoria-Gasteiz, Araba Spain

**Keywords:** Silver-Russell syndrome, Cri-du-chat syndrome, aCGH, Deletion

## Abstract

**Background:**

Silver-Russell Syndrome (SRS) is a rare growth-related genetic disorder mainly characterized by prenatal and postnatal growth failure. Although molecular causes are not clear in all cases, the most common mechanisms involved in SRS are loss of methylation on chromosome 11p15 (≈50%) and maternal uniparental disomy for chromosome 7 (upd(7)mat) (≈10%).

**Case presentation:**

We present a girl with clinical suspicion of SRS (intrauterine and postnatal growth retardation, prominent forehead, triangular face, mild psychomotor delay, transient neonatal hypoglycemia, mild hypotonia and single umbilical artery). Methylation and copy number variations at chromosomes 11 and 7 were studied by methylation-specific multiplex ligation-dependent probe amplification and as no alterations were found, molecular karyotyping was performed. A deletion at 5p15.33p15.2 was identified (arr[GRCh37] 5p15.33p15.2(25942–11644643)× 1), similar to those found in patients with Cri-du-chat Syndrome (CdCS). CdCS is a genetic disease resulting from a deletion of variable size occurring on the short arm of chromosome 5 (5p-), whose main feature is a high-pitched mewing cry in infancy, accompanied by multiple congenital anomalies, intellectual disability, microcephaly and facial dysmorphism.

**Conclusions:**

The absence of some CdCS features in the current patient could be due to the fact that in her case the critical regions responsible do not lie within the identified deletion. In fact, a literature review revealed a high degree of concordance between the clinical manifestations of the two syndromes.

**Electronic supplementary material:**

The online version of this article (10.1186/s12920-018-0441-z) contains supplementary material, which is available to authorized users.

## Background

Silver-Russel Syndrome (SRS, OMIM#180860) is a rare genetic imprinting disorder, initially described as an heterogeneous phenotype including intrauterine (IUGR) and postnatal growth retardation (PNGR) without catch-up growth, relative macrocephaly at birth, triangular face, body asymmetry, facial dysmorphic features and severe feeding difficulties [1, 2]. More recent case reports have led to the inclusion of low body mass index, hypoglycemia, motor and speech delay and psychosocial challenges as additional features (for a review, [3]). The incidence of the disease is not clear, reported estimates varying from 1/100,000 to 30/100,000 [3, 4]. Most cases of SRS are sporadic, with a low rate of familial cases that have been suggested to follow an autosomal dominant transmission pattern [5].

The wide variability of the clinical manifestations of SRS has led to the international recommendation to use the Netchine-Harbison SRS clinical scoring system (NH-CSS) [6], both for determining when SRS genetic testing should be run and when a clinical diagnosis of SRS should be given [3]. Although studies have failed to determine the underlying molecular mechanism in some cases, approximately 50% of the clinically-diagnosed SRS patients present alterations at 11p15.5, mainly hypomethylation at *H19/IGF2*:IG-DMR, while 10% of them show maternal uniparental disomy of chromosome 7 (upd(7)mat) [3]. In addition, there have been reports of a single maternally-transmitted *CDKN1C* activating mutation in five members of a four-generation family [7] and paternal *IGF2* inactivating mutations in another family and four unrelated patients [8–10]. Further, sequence variants of two non-imprinted genes (*HMGA2* and *PLAG1*) are also associated with SRS. Specifically, *HMGA2* variants have been described in one family and two sporadic cases [11, 12] and *PLAG1* mutations in a family and in one sporadic case [11]. For the remaining 40% of SRS patients who are negative for these alterations, molecular karyotyping is advised [3, 13, 14]. Two recent reviews have compiled all the reported chromosomal regions involved in SRS-like cases and suggested that the most frequently affected is 12q14, followed by 1q21, 4p16.3, 15q26, 17p13.3 and 22q11 [9, 15].

## Case presentation

The CARE guidelines were followed in reporting this case.

### Case report

**Fig. 1:**
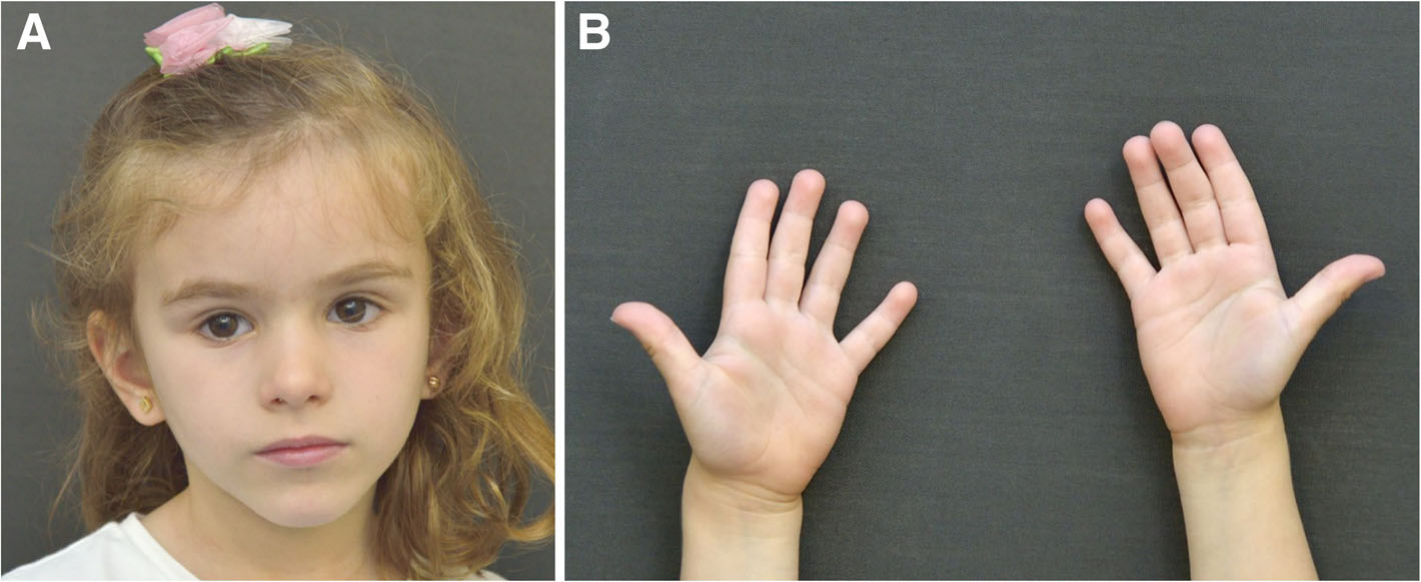
Clinical photograph of the patient. **a**: Front view of the face. Note the prominent forehead, triangular face, large eyes and narrow nasal bridge. **b**: Palmar view of the hand showing the small size and quadrangular fingertips

We present the case of a girl who is the third child of healthy non-consanguineous parents. A prenatal ultrasound test revealed a single umbilical artery with no other malformations. She was born at term, by vaginal delivery after induction due to fetal hypomobility, with a birth weight of 2450 g (*p* < 1), birth length of 47 cm (p3), and cranial perimeter of 33 cm (p10). No perinatal diseases were detected, except for one episode of transient neonatal hypoglycemia. In the neonatal period, no abnormal cry was noticed. She was monitored closely because of postnatal growth retardation in the absence of familial short stature (father’s height 180 cm, mother’s height 158 cm). At 22 months of age, she was referred for brain magnetic resonance imaging because of neurodevelopmental delay, and Arnold Chiari malformation type I and corpus callosum hypoplasia with mild ventriculomegaly were identified. A peculiar face with triangular shape was observed and height (78.5 cm, p3) and weight (9.7 kg, p7) were still delayed. Generalized hypotonia was still present. Surgery for Arnold Chiari I was performed, and subsequently, her motor development slightly improved. At the age of 4^9/12^, she was referred for clinical genetic assessment and SRS was suspected with a score of 4/6 on the NH-CSS: including IUGR, PNGR, prominent forehead and triangular face (Fig. 1a). At this age, she had a weight of 14.4 kg (p7), a height of 99.5 cm (p2) and a cranial perimeter of 49 cm (p15). Phenotypically, she also presented craniofacial disproportion, wide normal set rotated ears, a triangular face, large eyes and narrow nasal bridge (Fig. 1a), as well as small feet and hands with quadrangular fingertips (Fig. 1b). The 5th finger on each hand was shortened without clinodactyly (Fig. 1b); the great toe of each foot was wide and she had bilateral diastasis between the first and second toes. She also had a very smooth non-nasal voice.

### Molecular genetic studies

Genomic DNA was extracted from peripheral blood leukocytes using a commercial kit, following the manufacturer’s instructions (QiaAmp Blood Mini, Qiagen, Düren, Germany). Dosage and methylation analyses for chromosomes 11 and 7 were carried out by methylation-specific multiplex ligation-dependent probe amplification using the ME030-C3 and ME032-A1 kits, respectively (MRC-Holland, Amsterdam, The Netherlands) following the manufacturer’s recommendations. No alterations in methylation or copy number variations (CNV) were detected in either of these regions.

**Fig. 2:**
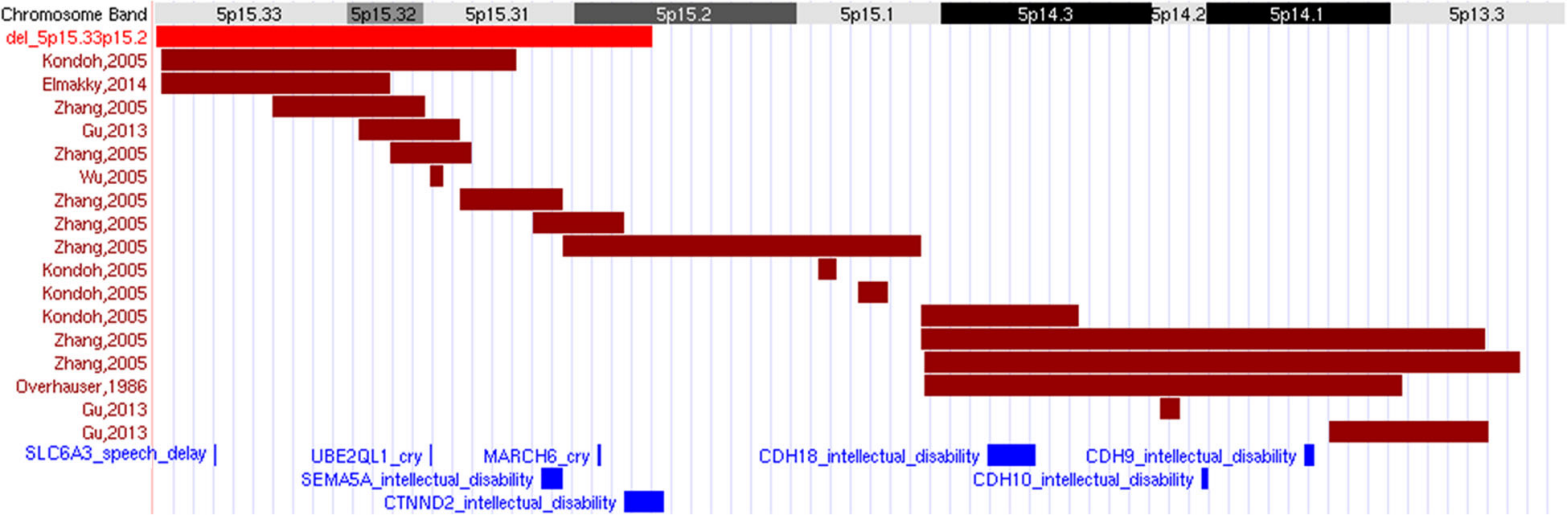
Schematic view of the short arm of chromosome 5, showing some described deletions and genes important in CdCS. The red bar at the top shows the deletion in the patient. Garnet bars are an indication of approximate regions deleted in several different patients described in literature, based on a recent review [35]. Some of the genes associated with the important features of CdCS are highlighted in blue

Subsequently, molecular karyotyping was performed using a 400 K microarray-based comparative genomic hybridization (aCGH) kit (G4448A, Agilent Technologies, Santa Clara, CA, USA). Slides were scanned on an Agilent SureScan C Microarray scanner and analyzed with Agilent CytoGenomics software, revealing a deletion on the short arm of chromosome 5, specifically at 5p15.33p15.2 (arr[GRCh37] 5p15.33p15.2(25942–11644643)× 1) (Fig. 2), within the region usually associated with Cri-du-chat syndrome (CdCS).

Fluorescence in situ hybridization (FISH) analyses were performed on metaphase cells and interphase nuclei using a Vysis CSF1R/D5S23, D5S721 FISH Probe Kit to confirm the presence of the deletion and its origin. The results showed one green signal (5p15) and two red signals (CSF1R probe) in the metaphases and nuclei of the index corresponding to a deletion of the 5p15 region (Additional file 1 Figure S1A, B), confirming the aCGH results. In accordance with the 2016 edition of the International System for Human Cytogenomic Nomenclature, the patient’s karyotype can be described as 46,XX,del(5)(p15.2).ish del(5)(p15.2)(D5S23-, D5S721-). The FISH pattern on chromosome 5 was normal in both parents (2G2R), confirming the de novo nature of the deletion (Additional file 1 Figure S1C-F).

### Clinical re-evaluation

A literature review showed us that there are some clinical features which are common to CdCS and SRS (Table 1). Keeping the results of this review and the genetic analysis in mind, clinical re-evaluation at 5 years 6 months of age confirmed some features suggestive of SRS (failure to thrive, retarded postnatal growth [height 104.5 cm (p4), weight 15.2 kg (p9), cranial perimeter 49.2 cm (p12)], prominent forehead) and some others of CdCS (slightly prominent chin with micrognathia); however, she did not have lip, palatal or mouth alterations; small hands with thin and long fingers; sandal gap deformity; or clinodactyly. The neurodevelopmental study revealed that she has not achieved the milestones for her chronological age (sitting without support and crawling at 12 months; walking independently at 25 months; very poor speech and verbal development with just 5–10 words at 2 years; and at the time of writing she still has neither bowel nor bladder control, is not able to read or write, and lacks fine motor skills). She is receiving psychological and learning support at school. She also has a high-pitched voice and dysphonia (possibly due to a defect in the larynx with vocal fold atrophy).

**Table 1:**
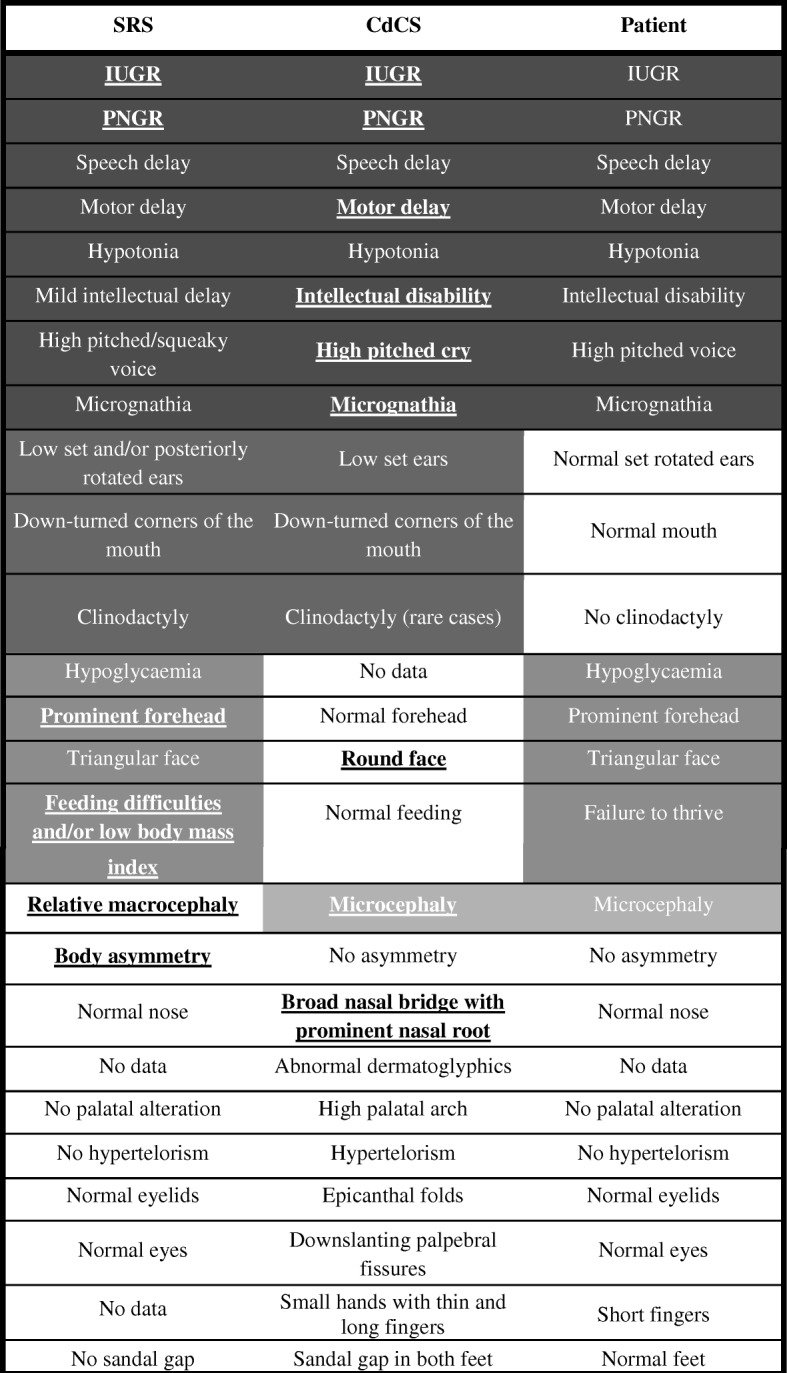
Clinical characteristics of Silver-Russell Syndrome (SRS) (Wakeling et al., 2017 [3]) and Cri-du-Chat Syndrome (CdCS) (Cerruti Mainardi, 2006 [21]; D M Church et al., 1995 [20]; D M Church et al., 1997 [22]) together with features observed in our patient

From darkest to lightest grey, the features are highlighted as follows: common features between (1) SRS, CdCS and the patient, (2) SRS and CdCS, (3) SRS and the patient and (4) CdCS and the patient. *IUGR* intrauterine growth retardation, *PNGR* post-natal growth retardation. Underlined features are those most specific to each syndrome

## Discussion and conclusions

Sometimes, clinical diagnosis of patients with syndromic manifestations is challenging due to either an absence of cardinal features or an overlap of characteristics between different disorders or a combination of both.

It is known that CdCS (OMIM#123450) is caused by deletions of heterogeneous size in the short arm of chromosome 5 [16]. Even though it is considered a rare disease, CdCS is one of the most common chromosomal deletion syndromes with an incidence ranging from 1:15,000 [17] to 1:50,000 live births [18]. In more than 80% of cases, the deletion is found to be de novo [19] and its extension seems to be closely related to the presence of certain phenotypic features [20].

The main clinical features at birth are: plaintive high-pitched monochromatic cry similar to the mewing of a cat (95.9%), which disappears after the neonatal period; microcephaly (mean head circumference, 31.8 cm) and low weight (mean weight, 2614 g). Other notable characteristics are: rounded face (83.5%), broad nasal bridge (87.2%), hypertelorism (81.4%), epicanthal folds (90.2%), downslanting palpebral fissures (56.9%), low-set ears (69.8%), micrognathia (96.7%), abnormal dermatoglyphics (92%), hypotonia and down-turned corners of the mouth (81.0%) [21]. CdCS patients also show severe psychomotor and intellectual disability, high palatal arch [20], speech delay, prenatal and postnatal growth delay, and low-set and/or poorly formed pinnae [22].

The majority of chromosome 5p deletions are associated with CdCS, but a del(5p) karyotype does not necessarily indicate CdCS [20, 23]. According to various different mapping studies using a strategy of “phenotype dissection”, some regions of the short arm of chromosome 5 have been associated with specific features which define certain aspects of the CdCS phenotype. Based on those studies, the critical factor responsible for the CdCS phenotype is considered to be the deletion located at 5p15.2, as haploinsufficiency of genes in this region is assumed to be associated with changes in facial features and severe mental retardation, as well as general CdCS features [19, 20, 22, 24]. Patients presenting the characteristic cat-like cry, the origin of the name of the syndrome, carry a deletion that includes a region proximal to 5p15.3 and distal to 5p15.2 [24, 25]. Different genes located within this region have been proposed to be responsible for the feature, namely, *UBE2QL1/FLJ28076* (5p15.31) and *MARCH6/TEB4* (5p15.2) [26]*.* Surprisingly, the deletion of the patient we report encompasses both candidate genes, but she did not have the classical cry, just a high-pitched voice. Coincidentally, there is another CdCS case described in the literature in which the patient did not have high pitched cry but did have a high-pitched voice. He had a 5p15.2 deletion, similar to our patient, and they share other clinical characteristics such as growth delay, slightly small chin, hypotonia and speech delay [22]***.***

The most distal part of 5p, the 5p15.3 band, has been reported to be related to speech delay [27]. In that region, more precisely at 5p15.33, studies have located the *SLC6A3* gene, which encodes an amine transporter responsible for dopamine reuptake. Curiously, an excess of dopamine has been associated with problems in speech due to its major role in fine motor movements and the fact that speech requires very accurate coordination of very diverse small muscles [28]. The patient we describe has speech problems, probably because of a defect in the in the larynx with vocal fold atrophy. Notably, the *SLC6A3* gene lies within the patient’s deletion, and that may explain the speech problems.

In addition, intellectual disability has been associated with a region at 5p15.2 [24], where the *CTNND2* gene is located [29]. This gene encodes a protein which plays a critical role in neural development, particularly in the formation and/or maintenance of dendritic spines and synapses [30]. In the same cytogenetic band, there is another region which seems to be responsible for the facial dysmorphism [19, 22, 31]. Further, alterations in *SEMA5A* (5p15.31) [32] and *CDH18* (5p14.3), *CDH10* (5p14.2) and *CDH9* (5p14.1) [33] also disrupt normal brain development; whereas autism spectrum and social communication disorders have been associated with the 5p14.1 cytogenetic band [34]. Like many other cases and as described above, our patient has intellectual disability, not having achieved the milestones for her age. Even though *CDH9, CDH10* and *CDH18* genes are supposed to be involved in intellectual disability, they may be not so critical because they do not lie within the region of the patient’s deletion. On the contrary, *CTNND2* and *SEMA5A* are in the deleted region and in consequence they may be responsible for this feature. On the other hand, autism is not one of the girl’s characteristics, perhaps due to the fact that the aforementioned region does not lie within the deletion. The *CDH9* gene is located in the band related to autism, and hence, it could be responsible for this feature apart from intellectual disability.

Some of these phenotypic features are in common with those of SRS (Table 1). In fact, the patient we describe has four out of the six cardinal characteristics [6] of SRS and lacks other important features of CdCS (high pitched cry, rounded face). What is more, the syndromes share some cardinal features, making it difficult to reach an accurate clinical diagnosis when they are present in a patient. On the other hand, according to the recent reviews [9, 15], ours is the first report of a patient with SRS carrying a deletion at 5q. As suggested above, the reason for the absence of these cardinal CdCS features could be that the chromosomal regions involved in these signs are not deleted in our patient. Nevertheless, as previously suggested by other authors [26], it is important to underline the great variability in each feature as each individual trait is not caused by alterations in a single gene. Detailed molecular analysis of more patients with well-established clinical features is necessary to identify the role of the genes responsible for the CdCS.

In brief, overlapping clinical manifestations of different disorders can lead to a misdiagnosis that could be avoided with more detailed molecular testing. Specifically, deletions at 5p should be considered in clinical SRS patients with negative results for chromosome 11 and 7 alterations.

On the other hand, given the importance of correlating the deleted regions at 5p and the clinical features associated with CdCS, some chromosomal maps have been developed. Nevertheless, more molecular karyotyping studies would help identify precise genomic coordinates responsible for each feature of the syndrome.

## Additional file

Additional file 1: **Figure S1.** FISH images of the index, her father and her mother. DX0164-I code is for the patient, DX0164-P for the father and DX0164-M for the mother. A, C, E pictures are from interphase nuclei and B, D, F from chromosomes in metaphase. One green signal (5p15) and two red signals (CSF1R probe) are visible in the index, suggesting a deletion of the 5p15 region. Both parents presented a normal result (2G2R) for FISH on chromosome 5. (TIF 235 kb)

## Abbreviations

5p-: Deletion on the short arm of chromosome 5
aCGH: Microarray-based Comparative Genomic Hybridization
CdCS: Cri-du-Chat Syndrome
CNV: Copy Number Variation
DMR: Differentially Methylated Region
FISH: Fluorescence In Situ Hybridization
IUGR: IntraUterine Growth Retardation
NH-CSS: Netchine-Harbison SRS Clinical Scoring System
PNGR: PostNatal Growth Retardation
SD: Standard Deviation
SRS: Silver-Russell Syndrome
upd(7)mat: Maternal uniparental disomy for chromosome 7
