# Supplementary figures and images for: Cri-du-chat syndrome mimics Silver-Russell syndrome depending on the size of the deletion: a case report
Source: BMC Med Genomics. 2018 Dec 27;11:124. doi: 10.1186/s12920-018-0441-z (PMC6307281; doi:10.1186/s12920-018-0441-z)

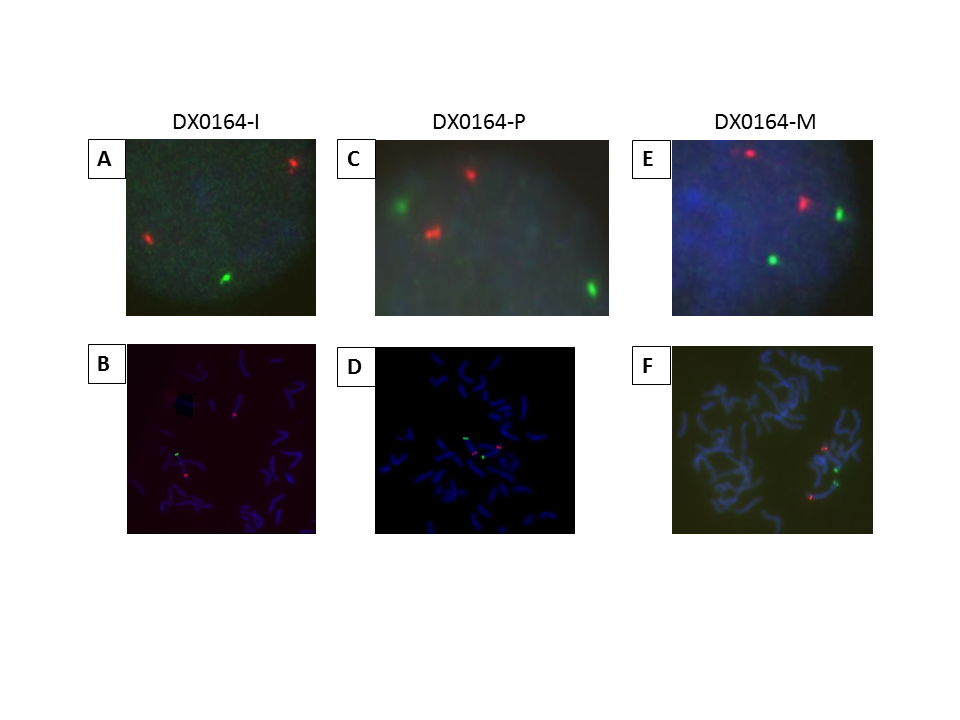

Supplement: Supplementary file 1 — Figure S1. FISH images of the index, her father and her mother. DX0164-I code is for the patient, DX0164-P for the father and DX0164-M for the mother. A, C, E pictures are from interphase nuclei and B, D, F from chromosomes in metaphase. One green signal (5p15) and two red signals (CSF1R probe) are visible in the index, suggesting a deletion of the 5p15 region. Both parents presented a normal result (2G2R) for FISH on chromosome 5. (TIF 235 kb) [file 12920_2018_441_MOESM1_ESM.tif]
